# Elevation of the mechanically-sensitive e protein emerin links nuclear mechanotransduction to tau-induced cytoskeletal remodeling in neurons

**DOI:** 10.1080/19491034.2026.2697135

**Published:** 2026-07-07

**Authors:** Claira Sohn, Sammy Pardo, Dana Molleur, Satvik R. Paduri, Morgan Lambert, Zachary Uttke, Erich J. Sohn, Morgan G. Thomas, Susan T. Weintraub, Bess Frost

**Affiliations:** a Department of Cell Systems and Anatomy, Barshop Institute for Longevity and Aging Studies, Glenn Biggs Institute for Alzheimer’s and Neurodegenerative Diseases, University of Texas Health San Antonio, San Antonio, TX, USA; b Department of Biochemistry and Structural Biology, University of Texas Health San Antonio, San Antonio, TX, USA; c Department of Molecular Biology, Cell Biology and Biochemistry, Center for Alzheimer’s Disease Research, Brown University, Providence, RI, USA; d Greehey Children’s Cancer Research Institute, University of Texas Health San Antonio, San Antonio, TX, USA

**Keywords:** Mechanotransduction, emerin, nucleus, cytoskeleton, tau, tauopathy, Alzheimer’s disease, actin

## Abstract

Neurodegenerative tauopathies, including Alzheimer’s disease, are neuropathologically defined by pathological tau deposition. While tau drives neurotoxicity by disrupting cytoskeletal, nucleoskeletal, and genomic architecture, cellular mechanisms mediating tau-induced dysfunction of the cytoskeleton and nucleoskeleton are incompletely understood. Here, we identify proteins with differing abundance in a cellular tauopathy model, iTau. Building upon previous findings that pathogenic tau reduces nuclear tension, we find elevated levels of emerin, a central regulator of nuclear mechanotransduction, in iTau neurons and tau mutant iPSC-derived neurons. Neuronal emerin overexpression drives neurotoxicity, increases filamentous actin (F-actin), and induces nuclear invagination, mimicking cellular phenotypes of tauopathy. Alterations in emerin binding partners reflect its cytosolic relocalization in iTau neurons, suggesting that pathogenic tau may impact nuclear mechanotransduction by altering emerin levels and localization. Overall, we identify emerin as a potential mediator of cytoskeletal and nucleoskeletal remodeling in tauopathy and provide a foundation for future studies of emerin function in neurons.

## Introduction

Cells of living organisms are subject to a variety of mechanical forces that influence cellular differentiation, size, morphology, and proliferation through a process termed mechanotransduction [1–3]. While mechanotransduction pathways have been primarily studied in non-neuronal cells, the brain is subject to acute and chronic changes in mechanical force due to blood flow through the vasculature, head injuries, aging, neurodegenerative processes, and other factors [4–6]. Studies in cultured rat cortical neurons suggest that neuronal networks have the capacity to adjust electrophysiological activity and cytoskeletal structure in response to mechanical force [7].

The nucleus is a central regulator of mechanotransduction [8–12]. Forces in the cytoplasm are transmitted to the nucleus through the nuclear membrane-spanning linker of nucleoskeleton and cytoskeleton (LINC) complex [11]. The LINC complex binds to actin and microtubules on the external face of the nuclear envelope and to lamin proteins along the internal face of the nuclear envelope. Lamin proteins assemble to form a meshwork of intermediate filaments that provide strength and structure to the nucleus. Lamina-associated polypeptide 2 (LAP2), emerin, and inner nuclear membrane protein Man1 (MAN1) are LEM-domain proteins that anchor the lamin nucleoskeleton [13]. Emerin responds dynamically to mechanical strain on the nucleus and regulates the induction of mechanosensitive genes [8,14,15].

A growing body of work suggests that nuclear form and function are disrupted in the context of various neurodegenerative disorders, including Alzheimer’s disease and related tauopathies. Mechanistically, pathological forms of tau are reported to drive nuclear invagination due to their effects on the actin cytoskeleton [16] and microtubule network [17]. Studies in *Drosophila* suggest that tau-induced increases in F-actin [17] are associated with clustering of Koi (LINC complex SUN domain protein) and decreased levels of B-type lamin; reducing MSP300 (LINC complex KASH-domain protein) rescues B-type lamin depletion and suppresses tau-induced neurotoxicity [16]. These findings support a model in which tau-induced elevation of F-actin alters mechanical force on the nucleus, leading to lamin loss and nucleoskeletal weakening [16]. Previous studies report that brains of patients with Alzheimer’s disease have reduced stiffness compared to age-matched controls, with regional stiffness further changing as the disease advances [18,19]. Based on these studies, alongside our previous finding that cultured cells harboring pathogenic tau have an overall decrease in nuclear tension [20], we hypothesized that pathways regulating nuclear mechanotransduction may be disrupted in tauopathy.

We find that pathological tau significantly alters the total abundance of proteins involved in cytoskeletal and nuclear function, including the mechanosensitive protein emerin. Studies in fibroblasts indicate that emerin is necessary for proper cytoskeletal and nuclear mechanics [14,21,22]. While there are few studies investigating the role of emerin in post-mitotic cells, recent findings suggest that emerin is elevated in neurons in response to sensory input and regulates activity-induced neuronal plasticity in mice [23]. We find that emerin overexpression in cultured neurons is sufficient to induce neurotoxicity, increase F-actin, and drive nuclear invagination. In the setting of tauopathy, we find that emerin is depleted from the nucleus and is enriched in the cytoplasm (presumably ER-associated), where it has increased interaction with cytoskeletal proteins and alters F-actin structure. Taken together, our findings suggest that elevation and redistribution of emerin from the nucleus to the cytoplasm disrupt the mechanical and signaling interface between the cytoskeleton and the nucleoskeleton, potentially compromising neuronal function in tauopathy.

## Results

### Proteomic profiling reveals cytoskeletal and nucleoskeletal regulators affected by pathogenic tau

The BE(2)-C iTau cellular model recapitulates the negative effects of tau on nuclear morphology [20] described in other laboratory models of tauopathy and in human disease [16,17,24–26]. iTau cells feature doxycycline-inducible expression of human tau carrying the frontotemporal dementia-associated [27] *MAPT* mutation R406W (tau^R406W^). We have previously used iTau cells to discover that pathogenic forms of tau decrease nuclear tension [20]. The inducible green fluorescent protein (iGFP) model, which does not accumulate disease-associated tau phosphoepitopes, serves as a control for transgenic protein overexpression. We leveraged iTau and iGFP cells in an unbiased approach to discover potential novel mechanisms underlying tau-induced cytoskeletal and nuclear dysfunction using data-independent acquisition (DIA) high-performance liquid chromatography (HPLC) mass spectrometry.

**Figure 1.**
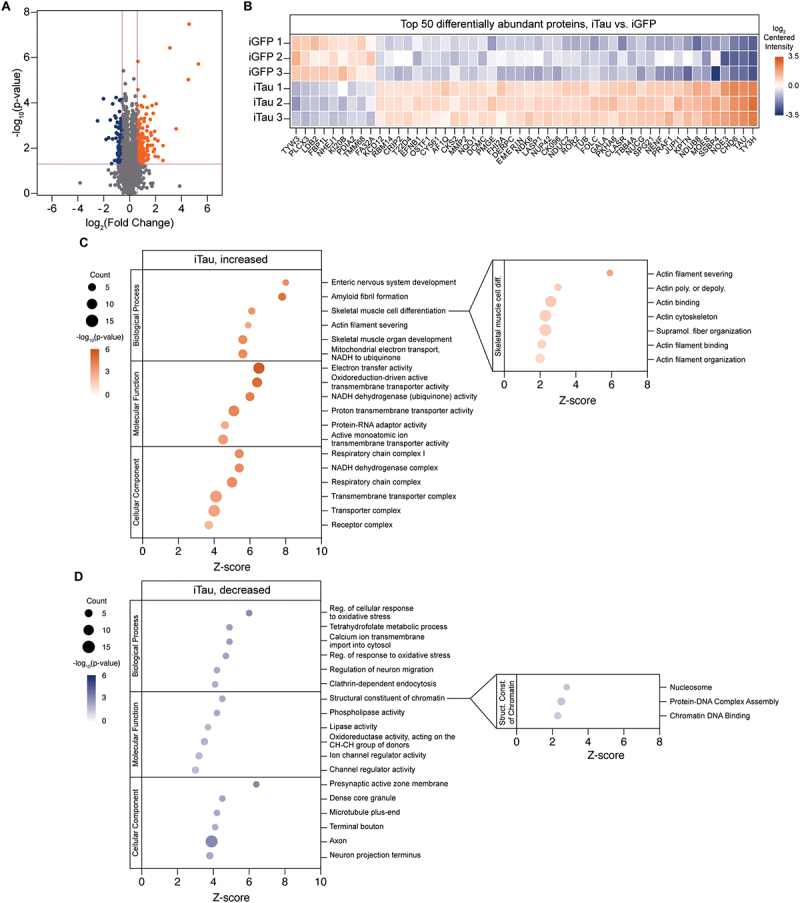
Proteomic profiling of iGFP and iTau cells reveals cytoskeletal and nucleoskeletal regulators affected by pathogenic tau. (A) Volcano plot showing differentially expressed proteins in iTau cells compared to iGFP based on mass spectrometry. (B) Top 50 significant proteins with the largest change in iTau cells. Metascape-based enrichment analysis of proteins that are significantly elevated (C) or depleted (D) in iTau cells. *n* = 3 biological replicates per group. Log_2_FC for significance is +/- 0.585 and *p* ≤ 0.05.

Proteomic analysis detects approximately 5,300 proteins per sample, with high group correlation among iGFP and iTau replicates (Supplemental Data 1). We identify 175 significantly upregulated and 115 significantly downregulated proteins in iTau vs. iGFP cells with a fold change of 1.5 or greater (Figure 1(A), the top 50 differentially abundant proteins are included in Figure 1(B)). Among proteins that are elevated in iTau cells, Metascape-based gene enrichment analysis [28] reveals a significant enrichment of proteins involved in metabolic pathways (Figure 1(C)), consistent with prior reports of disrupted lipid metabolism and mitochondrial function in tauopathy [29–33]. Relevant to our interest in regulators of cytoskeletal dysfunction in tauopathy, we find that components of the ‘skeletal muscle differentiation’ Gene Ontology (GO) term consist primarily of actin remodeling proteins. Consistent with prior work on nucleoskeletal instability [16] and heterochromatin decondensation [34] in tauopathy, proteins that are depleted in iTau cells show a significant enrichment for the GO term ‘structural constituent of chromatin’ (Figure 1(D)). Proteins involved in the oxidative stress response and neuronal compartments, including the axon, terminal bouton, and neuron projection terminus, are depleted in iTau cells, also consistent with previous work [35–37].

Of relevance to both cytoskeletal and nucleoskeletal stability, we find that emerin is among the top elevated proteins in iTau cells. Studies in non-neuronal cells identify emerin as a mechanically sensitive protein that responds to changes in cytoskeletal force and nuclear tension by shifting its localization from the nucleus to the outer nuclear membrane [8,38–40] and regulates mechanotransduction through interactions with the nucleo- and cytoskeleton [14,22]. While tau-induced elevation of emerin is highly relevant to our previous discovery that pathogenic forms of tau alter nuclear tension [20], little is known regarding the role of emerin as a mechanotransducer specifically in neurons. Given its significant upregulation in iTau cells, mechanosensitive properties, and dual role at the nucleo/cytoskeletal interface, we focused on emerin as a potential mediator of tau-induced changes in the cytoskeleton and nucleoskeleton.

### Emerin protein levels are elevated in neurons harboring pathological tau

**Figure 2.**
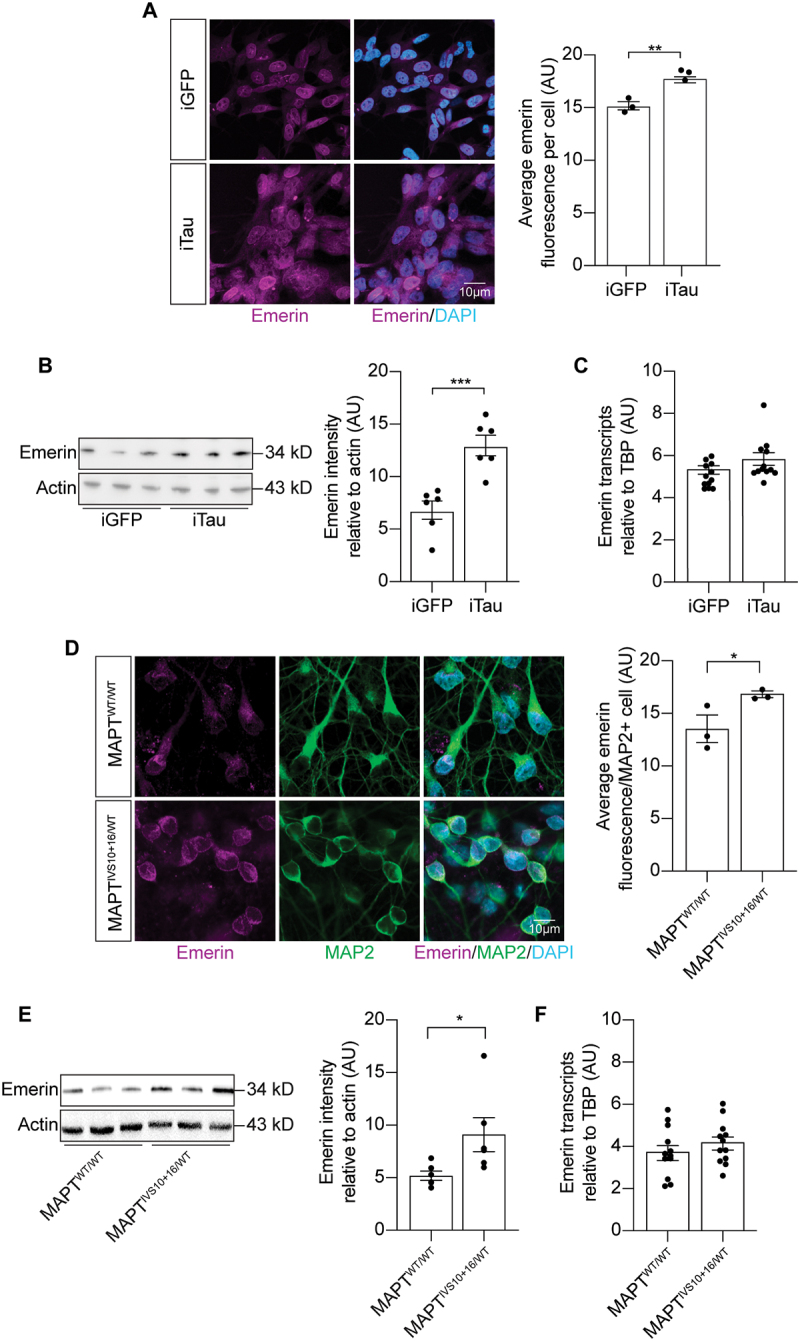
Emerin protein levels are elevated in two neuronal models of tauopathy. Significant increase in emerin in iTau neurons based on (A) immunofluorescence and (B) Western blotting after 1 week of retinoic acid-mediated neuronal differentiation and 24-hour doxycycline-mediated induction of tau or GFP expression. (C) Transcript levels of emerin are unchanged in iTau neurons based on ddPCR. Significant increase in emerin in *MAPT^IVS10+16/WT^* iPSC-derived neurons after 8 weeks of differentiation based on (D) immunofluorescence and (E) Western blotting. (F) Emerin transcripts are unchanged in *MAPT^IVS10+16/WT^* neurons based on ddPCR. *n* = 3 biological replicates per group for immunofluorescence and Western blotting; *n* = 12 biological replicates per group for ddPCR. t-test, *p ≤ 0.05, **p ≤ 0.01, ***p ≤ 0.001. Error bars indicate SEM.

As the BE(2)-C neuroblastoma cell line used to generate iTau and iGFP cells retains proliferative capacity and lacks key features of mature neurons, we induced cell cycle exit and neuronal differentiation by treating cells with 10 μM retinoic acid for 7 days. Following differentiation, we confirmed the neuronal identity of these cells based on expression of mature neuronal markers MAP2 and NeuN (Supplemental Figure S1(A,B)). iTau neurons robustly produce tau protein in response to 24 hours of exposure to doxycycline (Supplemental Figure S1(C)). We find that emerin protein is significantly elevated in iTau neurons based on immunofluorescence (Figure 2(A)) and Western blotting (Figure 2(B), Supplemental Figure S1(D)), with a fold change of 1.2 and 1.9, respectively. Interestingly, we find that emerin transcript levels are unchanged between iGFP and iTau neurons based on digital droplet PCR (ddPCR) (Figure 2(C)), suggesting a post-transcriptional mechanism underlying tau-induced elevation of emerin protein.

To determine if tau-induced emerin elevation is conserved in a cellular model that does not rely on tau overexpression, we utilized iPSC-derived neurons from a patient carrying a heterozygous *MAPT IVS10+16* mutation, which causes autosomal dominant frontotemporal dementia by altering tau splicing, along with its isogenic CRISPR-Cas9-corrected control [41]. Consistent with iTau-derived neurons, we find that emerin protein is elevated in *MAPT*^*IVS10+16*^ neurons after 8 weeks of differentiation based on immunofluorescence (Figure 2(D)) and Western blotting (Figure 2(E), Supplemental Figure S1(E)), with a fold change of 1.2 and 2.6, respectively. Similar to iTau neurons, emerin transcript levels are unchanged between *MAPT*^*IVS10+16*^ neurons vs. isogenic controls (Figure 2(F)). Taken together, these data demonstrate a tau-induced elevation of emerin that is conserved between different *MAPT* mutations and cellular models of tauopathy.

### Emerin regulates the actin cytoskeleton and lamin nucleoskeleton in BE(2)-C-derived neurons

**Figure 3.**
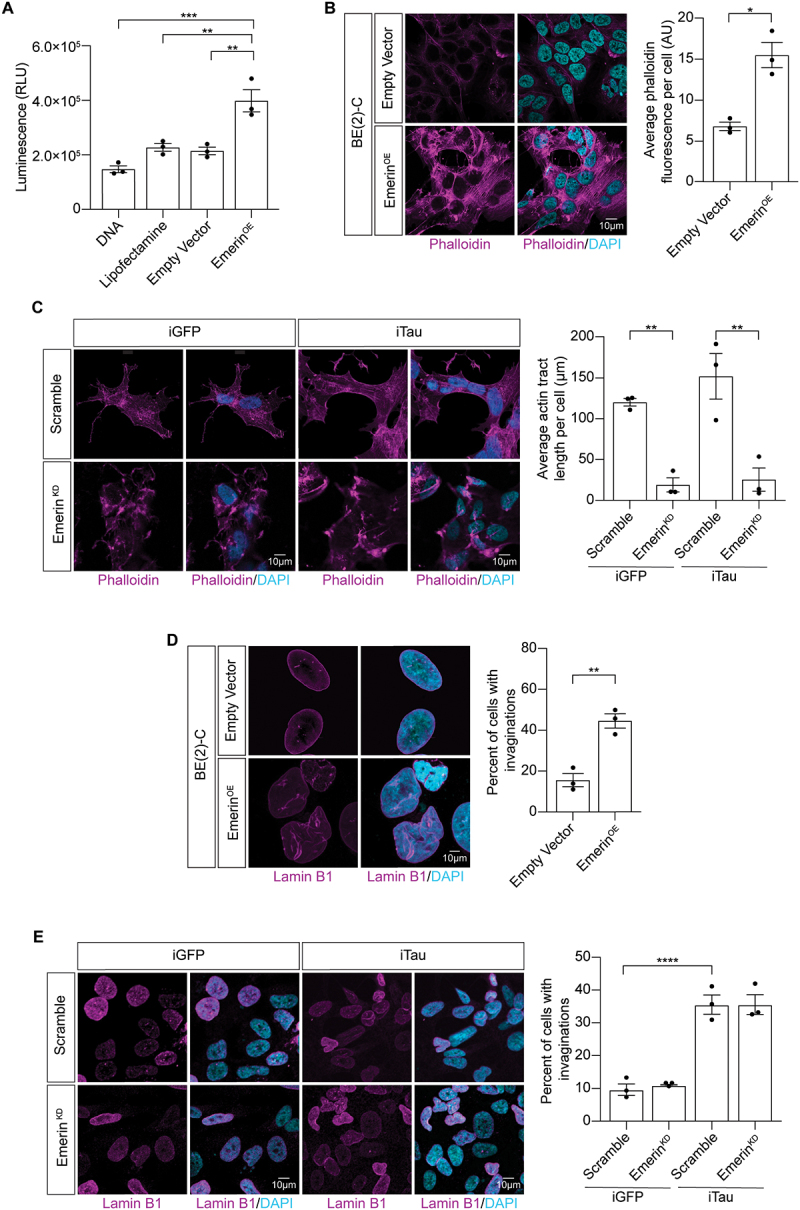
Emerin regulates the actin cytoskeleton and lamin nucleoskeleton in BE(2)-C-derived neurons. (A) Emerin overexpression in BE(2)-C-derived neurons causes neurotoxicity based on elevated levels of LDH in conditioned media. (B) Emerin overexpression in BE(2)-C-derived neurons causes an increase in F-actin levels based on phalloidin staining. (C) Lengths of F-actin tracts are significantly reduced in iGFP and iTau in response to RNAi-mediated emerin knockdown. (D) Emerin overexpression in BE(2)-C-derived neurons is sufficient to induce nuclear invagination based on lamin B1 immunofluorescence. (E) Nuclear invaginations are unchanged in iGFP and iTau neurons in response to RNAi-mediated emerin knockdown. *n* = 3 biological replicates per group, t-test or two-way ANOVA. *p ≤ 0.05, **p ≤ 0.01, ***p ≤ 0.001, ****p ≤ 0.0001. Error bars indicate SEM.

While *in vitro* studies and work in epithelial cells, HeLa cells, and mouse embryonic fibroblasts suggest that emerin preferentially binds to lamin A, and that disruption of B-type lamins does not alter the cellular localization or function of emerin [40,42–46], few studies have investigated the function of emerin in neurons. We transfected BE(2)-C-derived neurons with an emerin plasmid to determine if overexpression of emerin is sufficient to induce neurotoxicity. After validating robust emerin overexpression in BE(2)-C-derived neurons (Supplemental Figure S2(A,B)), we utilized a luminescent lactate dehydrogenase (LDH, released by cells upon plasma membrane damage) assay to assess potential emerin-associated neurotoxicity. We find that LDH levels are significantly elevated in conditioned media of emerin overexpressing BE(2)-C-derived neurons compared to negative controls, indicating that emerin overexpresssion is sufficient to drive toxicity in neurons (Figure 3(A)).

iTau neurons recapitulate features of human tauopathy, including increased incidence of nuclear envelope invagination [20] and elevated levels of F-actin (Supplemental Figure S2(C)). While *MAPT*^*IVS10+16*^ iPSC-derived neurons and cerebral organoids (aged to 20 weeks and 26 weeks, respectively) also have increased incidence of nuclear envelope invaginations [17,47], we find that both *MAPT*^*IVS10+16*^ and its isogenic control have high levels of nuclear invaginations at 8 weeks; the frequency of invaginations does not differ between conditions (Supplemental Figure S2(D)). Levels of F-actin are also equivalent between *MAPT*^*IVS10+16*^ and its isogenic control at 8 weeks of neuronal differentiation (Supplemental Figure S2(E)).

Given its important role in detecting and responding to forces on the nucleus, as well as its elevation in iTau and *MAPT*^*IVS10+16*^ neurons, we next determined if emerin mediates cytoskeletal structure and nuclear morphology in BE(2)-C-derived neurons. Based on phalloidin staining, we find that emerin overexpression is sufficient to increase overall levels of F-actin in neurons (Figure 3(B)). RNAi-mediated emerin knockdown (Supplemental Figure S2(F)) decreases the length of F-actin tracts in both iGFP and iTau neurons (Figure 3(C), Supplemental Figure S2(G)).

Considering previous evidence suggesting that tau-induced overstabilization of F-actin drives nuclear invagination [16], we next determined if emerin overexpression is sufficient to induce nuclear pleomorphism using immunofluorescence-based visualization of lamin B1. We detect a robust increase in nuclear invaginations in BE(2)-C-derived neurons overexpressing emerin (Figure 3(D)), indicating that emerin overexpression is sufficient to induce nuclear invagination in neurons. In contrast, RNAi-mediated emerin knockdown has no effect on the extent of nuclear invaginations in iTau neurons (Figure 3(E)), suggesting additional nuclear proteins (e.g., lamin B1) contribute to tau-induced nuclear pleomorphism. Taken together, our findings suggest that emerin overexpression has the capacity to drive neurotoxicity, F-actin stabilization, and nuclear invagination in neurons, and that a baseline level of emerin is required for the proper assembly or stabilization of neuronal F-actin.

### Pathogenic tau affects emerin interaction partners and nucleocytoplasmic localization

**Figure 4.**
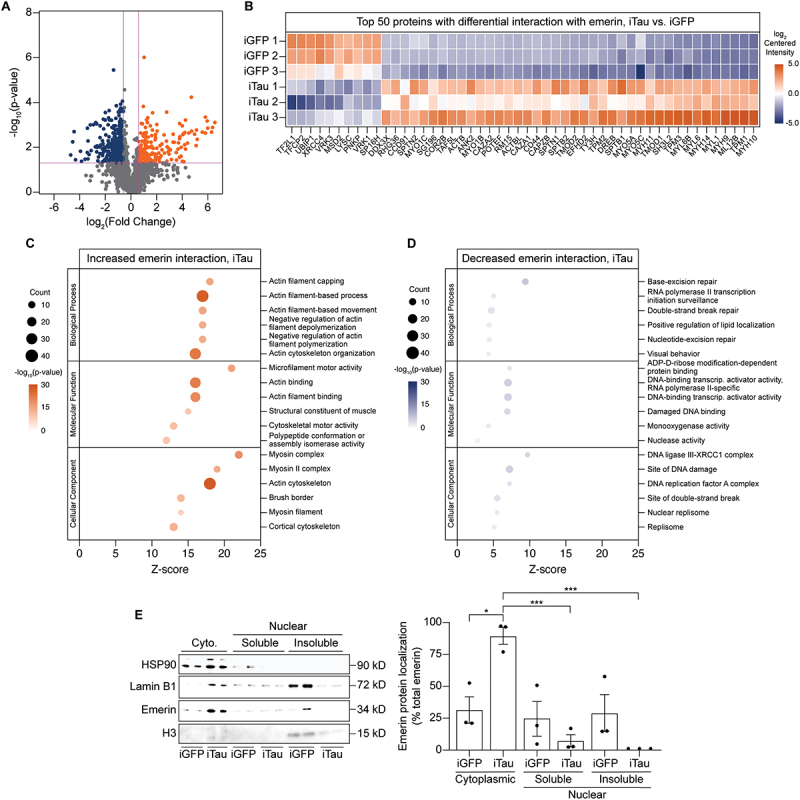
Emerin interactors and cellular localization are altered in iTau neurons. (A) Volcano plot shows the emerin interactome in iTau vs. iGFP neurons based on mass spectrometry. Log_2_FC for significance is +/- 0.585 and *p* ≤ 0.05, t-test. (B) Top 50 significant proteins with the largest change of emerin interaction in iTau vs. iGFP neurons. Metascape-based enrichment of proteins with increased (C) and decreased (D) emerin interaction in iTau neurons. (E) Cellular fractionation reveals cytoplasmic emerin elevation in iTau neurons, ANOVA, *p ≤ 0.05, ***p ≤ 0.001. *n* = 3 biological replicates per group.

Given that emerin is an inner nuclear membrane scaffolding protein that translocates to the outer nuclear membrane to alter actin polymerization in response to mechanical strain [48,49], we next determined whether pathogenic tau alters emerin binding partners. Immunoprecipitation of emerin from iGFP and iTau-derived neurons (Supplemental Figure S3(A)) followed by mass spectrometry identifies 3,800 proteins per sample, with high group correlation among replicates (Supplemental Data 2). We find that induced expression of pathogenic tau markedly alters proteins that interact with emerin in BE(2)-C derived neurons (Figure 4(A), the top 50 proteins with differential emerin interaction in iTau neurons are included in Figure 4(B)).

Using Metascape-based gene enrichment analysis [28], we find that proteins with significantly higher levels of emerin interaction in iTau neurons are enriched for GO terms associated with actin dynamics and myosin (Figure 4(C)). We detect significantly higher levels of emerin interaction with tau protein itself in iTau neurons, as well as actin capping proteins including tropomodulin 1 and 2 (TMOD1, TMOD2), F-actin-capping protein subunit alpha (CAZA), and F-actin-capping protein subunit beta (CAPZB). Proteins with significantly less emerin interaction in iTau neurons are enriched for GO terms associated with nuclear proteins, particularly those involved in DNA damage‑response pathways (Figure 4(D)). Consistent with previous studies reporting that pathogenic forms of tau cause nucleoskeletal destabilization [16,17,20,24,25] and heterochromatin decondensation [34,50], we find that emerin has decreased interaction with barrier to autointegration (BAF), a DNA‑binding protein that directly tethers emerin to chromatin [51], in iTau neurons.

Increased interaction between emerin and proteins that are enriched in the cytoplasm and decreased interaction between emerin and proteins that are enriched in the nucleus suggest that emerin may relocalize from the nucleus to the cytoplasm (presumably ER-associated) in the context of tauopathy. We performed cellular fractionation to directly determine if tau causes emerin to accumulate in the cytoplasm. While soluble levels of nuclear emerin are equal between iGFP and iTau neurons, insoluble nuclear emerin is undetectable in iTau neurons, and cytoplasmic emerin is significantly elevated (Figure 4(E), Supplemental Figure S3(B)). While overall levels of lamin B1 are unchanged in iTau vs. iGFP cells based on mass spectrometry, we find a decrease in insoluble nuclear lamin B1 in iTau neurons, consistent with nucleoskeletal instability and with previous reports of lamin B1 localization in the cytoplasm in neurons [52]. Taken together, these analyses suggest that the intracellular mechanical environment is significantly disrupted in tauopathy and that pathological tau causes emerin to accumulate in the cytoplasm, where it regulates cytoskeletal structure.

## Discussion

Extensive evidence from multiple model systems suggests that various forms of pathogenic tau disrupt the neuronal cytoskeleton and nucleoskeleton [16,17,20,24–26,53–55]. Mechanistically, studies in *Drosophila* demonstrate that pathological tau causes overstabilization of F-actin [54], which disrupts the LINC complex and destabilizes the lamin nucleoskeleton, ultimately contributing to nuclear invagination, blebbing, and neuronal death [16]. Complementary work in iPSC-derived neurons reveals that tau pathology impairs the microtubule network, which also contributes to nuclear invaginations and blebbing [17]. In primary neurons, optogenetic induction of tau oligomerization deforms the nuclear envelope and promotes tau multimer interactions with many nuclear proteins, including A- and B-type lamins, LBR, Nup93, and Nup133 [25].

Given the well-established effects of tau on the cyto- and nucleoskeleton, alongside our previous finding that pathogenic forms of tau reduce nuclear tension [20], we were interested to find that the mechanically sensitive protein emerin is elevated in two cellular models that harbor different *MAPT* mutations. While our initial proteomic analyses utilized cells that overexpress tau harboring an *R406W* missense mutation, emerin protein is also elevated in iPSC-derived neurons carrying the *IVS10+16* splice‑site mutation. Both mutations cause autosomal dominant frontotemporal dementia [27]; *IVS10+16* shifts the splicing ratio to favor the production of tau with four microtubule‑binding domains rather than altering the amino acid sequence of the tau protein itself. These data suggest that tau-induced emerin elevation is a general response to the presence of pathogenic tau species rather than a specific consequence of a particular *MAPT* mutation.

e find that iTau neurons faithfully recapitulate nuclear envelope invagination and overstabilization of F-actin reported in tau-based model systems and in postmortem human tau-affected brain tissue. While an increase in nuclear envelope invagination has been previously reported in *MAPT*^*IVS10+16*^ iPSC-derived neurons aged to 20 weeks [17] and in *MAPT*^*IVS10+16*^‑derived cerebral organoids aged to 26 weeks [47], we do not detect a significant difference in invaginations between *MAPT*^*IVS10+16*^ neurons and isogenic controls aged to 8 weeks. We speculate that this discrepancy is due to the relatively fetal-like developmental state of iPSC-derived neurons at 8 weeks [56,57]. Consistent with the high frequency of nuclear invagination in developmentally immature cells [58], we find that nearly half of all neurons harbor nuclear invaginations in iPSC-derived neurons at 8 weeks. Levels of F-actin are also unchanged between *MAPT*^*IVS10+16*^ and isogenic controls. Developing neurons maintain a higher ratio of G- to F-actin to allow for plasticity required for neurite extension and synaptic pruning, while mature neurons possess a more stable F-actin cytoskeleton [59]. These data suggest that the aberrant forces exerted by elevated emerin may be mechanically buffered in the structurally dynamic environment of an 8-week-old iPSC-derived neuron.

Together with previous work demonstrating that pathogenic forms of tau decrease nuclear tension [20], our findings suggest that tau pathogenicity impairs neuronal mechanotransduction. By driving the mislocalization of emerin, a major factor in sensing and responding to mechanical forces upon the nucleus, from the nucleus to the cytoplasm, pathogenic forms of tau may uncouple the nucleoskeleton from its LINC-interacting cytoskeletal inputs. Given the interdependency between actin, the LINC complex, lamin proteins, and emerin, this disruption likely has consequences beyond altered mechanotransduction. Consistent with previous work suggesting that pathogenic forms of tau cause decondensation of constitutive heterochromatin [34], we detect decreased interaction between emerin and BAF in iTau neurons, suggesting that emerin dysfunction in tauopathy also disrupts the physical tethering of heterochromatin to the nuclear lamina. As emerin serves as a scaffold for an array of nuclear proteins beyond BAF, including various transcription factors [60], we speculate that nuclear signaling complexes are significantly altered in tauopathy. Decreased interaction between emerin and a suite of essential DNA repair enzymes, including XRCC4, XRCC1, LIG3, and PNKP, in iTau neurons further suggests that emerin dysfunction may affect the ability of neurons to respond to DNA damage.

The elevation of emerin observed in tau-based models presents an interesting contrast to X-linked Emery-Dreifuss Muscular Dystrophy (EDMD), which is characterized by a loss of emerin function [61]. While loss of emerin in EDMD primarily impacts skeletal and cardiac muscle through weakened structural integrity of the nucleus [62], our findings suggest that emerin elevation may be equally detrimental to post-mitotic neurons. In tauopathy, the potential loss of nuclear emerin coupled with enrichment of cytoplasmic emerin may cause a dual hit to neuronal integrity by reducing the ability of neurons to maintain genomic architecture and impeding the cytoskeletal plasticity required for synaptic function and intracellular transport.

To date, most functional studies of emerin have been conducted in the context of non-neuronal cells. Our analyses provide new insight into the function and negative consequences of emerin elevation in neurons and identify emerin as a candidate mediator of mechanical dysfunction in tauopathy. This work provides a strong foundation for future studies exploring how tau pathology alters the mechanical landscape in neurons and highlights emerin as a potential effector of tau-induced toxicity.

## Materials and methods

### BE(2)-C cell culture

Human neuroblastoma cells (BE(2)-C; ATCC #CRL-2268) were cultured in a 1:1 mixture of Eagle’s Minimum Essential Medium (EMEM) and F12 medium supplemented with 10% tetracycline-free FBS and 1% penicillin-streptomycin. As previously described [20], stable cell pools of BE(2)-C_MAPT-R406W and BE(2)-C_GFP were generated by transducing BE(2)-C cells with PLIX_403_MAPT-R406W or PLIX_403_GFP lentiviral particles, followed by selection with 2 µg/mL puromycin. *MAPT* cDNA (NM_005910.6) with a single mutation (1216C > T) and *GFP* cDNA were synthesized and inserted into the doxycycline-inducible lentiviral vector PLIX_403 (Addgene #41395). The MD Anderson Functional Genomics Core Facility generated lentiviral particles. Expression of MAPT-R406W or GFP was induced with 1 µg/mL doxycycline hyclate dissolved in DMSO [20].

For differentiation into neurons, cells were plated at 5.0 × 10^4^ in 6-well plates for 24 hours in a 1:1 mixture of EMEM and F12 medium supplemented with 10% tetracycline-free FBS and 1% penicillin-streptomycin. From days two to six, cells were cultured in EMEM, 1% tetracycline-free FBS, 1% penicillin-streptomycin, and 10 μm retinoic acid (#R2625, Sigma-Aldrich). Cells were fed every other day until day six, at which point GFP or tau^R406W^ expression was induced as described above.

### iPSC culture

Human iPSC lines were obtained from the Tau Consortium cell line collection (https://www.neuralsci.org/tau) [41]. iPSCs were maintained in complete mTeSR1 medium (#85850, STEMCELL Technologies) on Corning® Matrigel® hESC-Qualified Matrix (#354277, Corning) and passaged every 3–5 days using ReLeSR™ (#05872, STEMCELL Technologies). To generate neural aggregates, iPSCs were plated at 3.0 × 10^6^ cells per well in Nunclon Sphera 96 U-bottom plates (#174929, Thermo Fisher Scientific) in STEMdiff™ Neural Induction Medium (NIM) (#05835, STEMCELL Technologies) supplemented with 1 μL/mL ROCK inhibitor Y-27632 (#72302, STEMCELL Technologies). ROCK inhibitor was removed after 24 hours. Half-media changes (75 μL) were performed from days 2–6, using fresh NIM supplemented with dorsomorphin (#P5499, Sigma-Aldrich) and SB-431542 (#1614, Tocris).

On day 6, aggregates were washed with DMEM/F12 and transferred to Matrigel-coated 6-well plates (32 aggregates per well) to generate neural rosettes. Rosettes were fed daily with NIM for 3–7 days until flattening and neural cluster formation were observed. Rosettes were harvested using Neural Rosette Selection Reagent (#05832, STEMCELL Technologies), incubated for 35–40 minutes, rinsed with DMEM/F12, and collected by centrifugation at 750 rpm for 3 minutes. Pellets were resuspended in fresh NIM.

Cells were expanded to ~80–90% confluence and passaged onto 6-well plates and glass-bottom dishes to generate neural progenitor cells (NPCs). NPC identity was confirmed by immunofluorescence for Nestin, Pax6, Oct4, and Sox2. NPCs were then plated for neuronal maturation.

For neuronal differentiation, cells were maintained in BrainPhys™ Neuronal Medium (#05790, STEMCELL Technologies) supplemented with NeuroCult™ SM1 (#05711), N2 Supplement-A (#07152), 20 ng/mL BDNF (#78005), 1 mM dibutyryl-cAMP (#73886), and 200 nM ascorbic acid (#72132). Neurons were fed three times weekly (Monday, Wednesday, Friday) for 8 weeks, at which point they were utilized for downstream analyses.

### Mass spectrometry sample preparation

One T75 (global proteomics) and 12 T75s (emerin interactome analysis) were cultured to 95% confluency per replicate. Cells were washed with dPBS prior to harvesting, after which 0.25% trypsin was added to each flask for 3 minutes. 9 mL of F12 and 1 mL of FBS were added to the flask to dilute the trypsin, and cells were centrifuged for 5 minutes (1,000 rpm, room temperature). Cell pellets were washed with 10 mL of dPBS and centrifuged for 5 minutes (1,000 rpm, room temperature) to re-pellet. Cell pellets were flash frozen and stored at −80°C.

Cell samples were randomized for preparation and DIA-MS analysis. Cells were lysed in a buffer containing 5% SDS/50 mM triethylammonium bicarbonate (TEAB) in the presence of protease and phosphatase inhibitors (#78440; ThermoFisher Scientific) and nuclease (#88700, ThermoFisher Scientific). Aliquots of the lysates containing 100 µg of protein (#R33200, ThermoFisher Scientific) were mixed with a buffer containing 10% SDS/50 mM TEAB, reduced with tris(2-carboxyethyl)phosphine hydrochloride (TCEP), and alkylated in the dark by iodoacetamide. After quenching with dithiothreitol, 12% phosphoric acid solution was added to each sample, and the mixtures were applied to S-Traps (mini; ProtiFi) for tryptic digestion (#V5111; Promega) in 50 mM TEAB. Peptides were eluted from the S-Traps sequentially with 50 mM TEAB, 0.2% formic acid, and 0.2% formic acid in 50% aqueous acetonitrile. Pooled eluates were dried by vacuum centrifugation, redissolved in the starting HPLC mobile phase (3% B, see Mass spectrometry analysis (DIA-MS) and data processing), and quantified using Pierce™ Quantitative Fluorometric Peptide Assay (#23290, ThermoFisher Scientific). Sample injections for mass spectrometry analysis were 2 µg of peptides in 5 µL.

To extract protein lysate for emerin immunoprecipitation, cell pellets were placed on ice in 500 μL of ice-cold lysis buffer consisting of 50 mM Tris-HCl pH 7.4, 300 mM NaCl, 0.3% v/v Triton X-100, 5 mM EDTA, 1 mM DTT, 100 μM PMSF, 1 μg/mL pepstatin A, and 1X Halt protease inhibitor cocktail (#78430, ThermoFisher Scientific). Once thawed, cell pellets were resuspended by vortexing. Samples were incubated on ice for 10 minutes, sonicated on ice for a total processing time of 10 seconds (intervals of 0.5 seconds ON/30 seconds OFF), and centrifuged for 30 minutes (16,000xg, 4°C) to pellet insoluble material. Each sample was quantified via Pierce BCA assay (#23225, ThermoFisher Scientific) and then diluted to ~7 mg/mL and frozen at −80°C.

For emerin immunoprecipitation, 50 μL per sample of Dynabeads™ Protein-G (#10003D, ThermoFisher Scientific) was washed with 500 μL of PBS +0.05% Tween 20. The tubes were then placed in a magnetic tube holder, and the wash buffer was aspirated. Washed Dynabeads were incubated at 4°C on a rotator with 1 μg/μL primary antibody (ab40688, Abcam) in PBS for 6 hours. After unbound antibody was removed, 200 μL of protein lysate was added and incubated at 4°C on a rotator overnight. Dynabeads were then washed three times in 0.05% PBS Tween 20, and protein was eluted in 15 μL of 0.5 mM glycine pH 2.8. The elution was added to 15 μL of 2x Laemmli with β-mercaptoethanol and boiled at 70°C for 10 minutes. Samples were stored at −80°C.

Samples from the emerin co-immunoprecipitation experiment were blocked by replicate and randomized within each replicate for preparation and DIA-MS analysis. The eluates (30 µL) were mixed with 12% phosphoric acid solution and 10% SDS/50 mM TEAB, applied to S-Traps (micro; Protifi), reduced/alkylated with a mixture of 10 mM TCEP/25 mM 2-chloroacetamide in 50 mM TEAB, and then digested with trypsin. Peptide quantitative analysis was not conducted. Equal volumes of digest were used for DIA-MS analysis.

### Mass spectrometry analysis (DIA-MS) and data processing

DIA-MS was conducted on an Orbitrap Fusion Lumos mass spectrometer (Thermo Scientific). On-line HPLC separation was accomplished with an RSLC NANO HPLC system (Thermo Scientific/Dionex). Conditions for analysis of the cell lysates were as follows: column, PicoFrit™ (New Objective; 75 μm i.d.) packed to 15 cm with C18 adsorbent (Vydac; 218 MS 5 μm, 300 Å); mobile phase A, 0.5% acetic acid (HAc)/0.005% trifluoroacetic acid (TFA) in water; mobile phase B, 90% acetonitrile/0.5% HAc/0.005% TFA/9.5% water; gradient 3 to 42% B in 120 min; flow rate, 0.4 μL/min. Separation conditions for peptides from the co-IP experiments were as follows: column, PepSep (Bruker; ReproSil C18, 15 cm ×150 µm, 1.9 µm beads); mobile phases A and B and gradient, same as above; flow rate, 800 nL/min. Peptides from cells were analyzed independently from the co-IP experiment. Separate pools were made of the digests of the cell lysates (0.4 µg/µL) and the co-IP digests (equal volumes of each). Aliquots of the pools (cells, 2 µg in 5 µL; co-IP digests, equal proportion in 5 µL) were analyzed using three stages of gas-phase fractionation (395–605 m/z, 595–805 m/z, 795–1005 m/z, staggered) and 4-m/z windows (30k resolution for precursor and product ion scans, all in the orbitrap). The resulting three data files were used to create an empirically corrected DIA chromatogram library [63] by searching against a Prosit-generated predicted spectral library [64] based on the UniProt Human protein database [UniProt_Human_ref 9606_20220216 (20,588 sequences; 11,394,277 residues)]. MS data for the experimental samples were acquired using 8-m/z windows (400–1000 m/z; staggered; 30k resolution for precursor and product ion scans) and searched against the chromatogram library. *Scaffold DIA* (Proteome Software; cells, v3.3.1; co-IP, v3.4.1) was used for all DIA-data processing: fixed modification, cysteine carbamidomethylation; proteolytic enzyme, trypsin with one missed cleavage allowed; peptide mass tolerance, ±10.0 ppm; fragment mass tolerance, ±10.0 ppm; charge states, 2+ and 3+; peptide length, 6–30. Files were separately processed for cells and co-IP digests. Peptides identified in each sample were filtered by *Percolator* [65] to achieve a maximum FDR of 1%. Individual search results for each sample type were combined, and peptide identifications were assigned posterior error probabilities and filtered to an FDR threshold of 1% by *Percolator*. Peptide quantification was performed by *Encyclopedia* [63] based on the three to five highest quality fragment ions. Only peptides that were exclusively assigned to a protein were used for relative quantification.

### Western blotting

Cells were harvested at ~90% confluency using RIPA with 1x Halt protease inhibitor (#78430, ThermoFisher Scientific) and incubated at 4°C for 30 minutes with gentle rocking. Cell lysates were centrifuged for 20 minutes (12,000 rpm, 4°C). A Pierce BCA assay (#23225, ThermoFisher Scientific) was performed on the supernatants to quantify protein concentration. Protein lysates were boiled in 2x Laemmli buffer for 5 minutes, centrifuged for 1 minute (12,000 rpm, room temperature) then loaded onto a 4–20% SDS – PAGE gel with 20 µg of protein loaded per well. Equal loading was assessed by Ponceau S staining of nitrocellulose membranes after transfer. Membranes were blocked in PBS containing 0.05% Tween 20 and 2% milk then incubated with primary antibodies overnight at 4°C (Supplemental Table S1). After washing, membranes were incubated with HRP-conjugated secondary antibodies for 2 hours at room temperature. Blots were developed with an enhanced chemiluminescent substrate. Densitometry was performed using ImageJ.

### Immunofluorescence

Cells were plated in 12-well plates on 20 mm coverslips prior to staining. Cells were fixed in 100% ice-cold methanol at room temperature for 5 minutes and then washed three times with 0.01% Tween 20 in PBS for 5 minutes per wash. After washing, cells were permeabilized in 0.01% PBS Triton X-100 with 1% bovine serum albumin (BSA) for 15 minutes at room temperature, washed three times with 0.01% Tween 20 in PBS, and then blocked for 30 minutes in PBS with 1% BSA and 0.01% Tween 20. Primary antibodies were diluted in 1% BSA and incubated with cells overnight at 4°C (Supplemental Table S1). The following day, cells were washed with 0.01% Tween 20 in PBS and then incubated with secondary antibody for 1 hour at room temperature. After washing, cells were incubated with 1X DAPI for 2 minutes to stain nuclei and then mounted onto glass slides with Vectashield (#H-1000–10, Vectorlabs). To visualize actin, cells were stained with Acti-Stain 555 Phalloidin (#PHDH1-A, Cytoskeleton) for 30 minutes before DAPI staining (Supplemental Table S2). Cells were visualized by confocal microscopy (Zeiss LSM 880 or Zeiss LSM 980).

### Image analysis

The maximum projection from Z-Stacks was used for quantification of nuclear invaginations. To calculate the percentage of nuclei containing nuclear invaginations, 100 cells per replicate were scored for the presence of invaginations. Quantification of average fluorescence of emerin and phalloidin in iGFP/iTau neurons was performed using ImageJ by quantifying the average mean intensities based on a mask around emerin or F-actin. The mean intensity was divided by the number of nuclei in each frame, excluding any fluorescence signal or nuclei on the edges. Quantification of emerin in *MAPT*^*IVS10+16*^ neurons was completed by measuring mean intensities within MAP2-positive neurons. Lengths of F-actin tracts were quantified in ImageJ using the plugin Tubeness, followed by Ridge detection. Combined, these two plugins allow the quantification of tract length in pixel units, which were converted to microns (μm) using the set scale feature. Total F-actin tract length per frame was divided by the total number of nuclei present within the frame to calculate the average actin tract length per cell, which was then divided by a pixel-to-micron conversion factor. Values for at least five frames per condition were averaged for each replicate.

### ddPCR

One 6-well plate was collected per biological replicate using TRIzol (#15596026, ThermoFisher Scientific) according to the manufacturer’s protocol. RNA quantities were measured using a Nanodrop8000 spectrophotometer (ThermoFisher Scientific). Equal quantities of RNA were added to a reverse transcription reaction (#4368814, ThermoFisher Scientific). Equal quantities of cDNA were loaded into QX200 Droplet PCR System (Bio-Rad). Probes for emerin and TATA-box binding protein (TBP) were predesigned by Bio-Rad.

### Transfection of emerin siRNA and emerin overexpression vector

Overall, 1% penicillin-streptomycin was removed from the media on day four of differentiation for siRNA transfection and day five for emerin overexpression. Media was replaced with Gibco Opti-MEM reduced serum medium (#31985088, ThermoFisher Scientific). Cells were transfected in a 6-well plate with 100 nM of emerin Silencer® Select siRNA (#4392420, ThermoFisher Scientific, Assay ID S4647 or S4645) or Silencer® Select Negative Control No.1 (#4390843, ThermoFisher Scientific) with 9 μL/well of Lipofectamine RNAiMAX Transfection Reagent (#13778030, ThermoFisher Scientific) following the manufacturer’s protocol. For emerin overexpression, cells were transfected with 4 μg of emerin plasmid or empty vector (Addgene ID: 253,815 and 253,816) with 9 μL/well of Lipofectamine 2000 (#11668019, ThermoFisher Scientific). Cells were incubated with siRNA/Lipofectamine or emerin/Lipofectamine for 24 hours and 6 hours, respectively. Transfection media was replaced by EMEM, 1% tetracycline-free FBS, 10 μm retinoic acid (#R2625, Sigma-Aldrich), and 1 µg/mL doxycycline hyclate in DMSO. Cells were collected for experimentation on day seven.

### Statistical analyses and rigor

The false discovery rate (FDR) was set to 1.0% for mass spectrometry analysis. Samples with one or more missing values, and any proteins that had a peptide count of less than two were excluded. A *p* ≤ 0.05, and a Log_2_FC of −0.585 and 0.585 were considered significant. Correlations among and between samples were determined using the Pearson correlation coefficient. Proteins that were significantly up- and down-regulated or had a significant increase or decrease in interaction with emerin were entered into Metascape.org for Enrichment analysis. The custom analysis function in Metascape was used to select for Biological Process (GO), Cellular Component (GO), and Molecular Function (GO). The analysis species was set to *Homo sapiens*. All plots were created using R Studio.

For immunofluorescence, samples were randomized during image acquisition and were analyzed by ImageJ. All cell replicates reflect different passage numbers from different flasks. Each experiment was completed in triplicate on different days. Western blots were analyzed by band densitometry using ImageJ. For experiments with more than two groups, statistical significance was assessed by one-way ANOVA with Tukey’s multiple comparisons test. Two-group comparisons were analyzed using a two-tailed Student’s t-test. Normality and homogeneity of variance were assessed before parametric testing. A *p*-value < 0.05 was considered significant. GraphPad Prism was used for the analysis of immunofluorescence, Western blotting, and ddPCR experiments.

## Supplementary Material

2026 Sohn Nucleus Supplemental Data 2.xlsx

Sohn Supp Fig 2.tif

2026 Sohn Nucleus Supplemental Data 1.xlsx

Sohn Supp Fig 1.tif

2026 Sohn Nucleus Supplemental Information.docx

## Data Availability

Mass spectrometry datasets will be made publicly available through the ProteomeXchange upon acceptance of the manuscript.

## Significance statement

Cells experience and respond to diverse mechanical forces that shape their morphology, function, and survival through a process termed ‘mechanotransduction.’ While well studied in non-neuronal cells, neuronal mechanotransduction remains poorly understood despite exposure of the brain to vascular flow, movement, injury, and disease. We identify the mechanosensitive protein emerin as a key regulator of nuclear mechanotransduction in neurons. Emerin overexpression is sufficient to drive neurotoxicity, increase filamentous actin, and induce nuclear invagination, revealing a novel function for emerin in neurons. In cellular models of tauopathy, emerin is elevated and relocalizes from the nucleus to the cytoplasm (presumably ER-associated), where it alters cytoskeletal structure. These findings establish emerin as a mechanosensitive regulator in neurons and link disrupted nuclear neuronal mechanotransduction to neurodegeneration.
