# Supplementary figures and images for: Elevation of the mechanically-sensitive e protein emerin links nuclear mechanotransduction to tau-induced cytoskeletal remodeling in neurons
Source: Nucleus. 2026 Jul 7;17(1):2697135. doi: 10.1080/19491034.2026.2697135 (PMC13349007; doi:10.1080/19491034.2026.2697135)

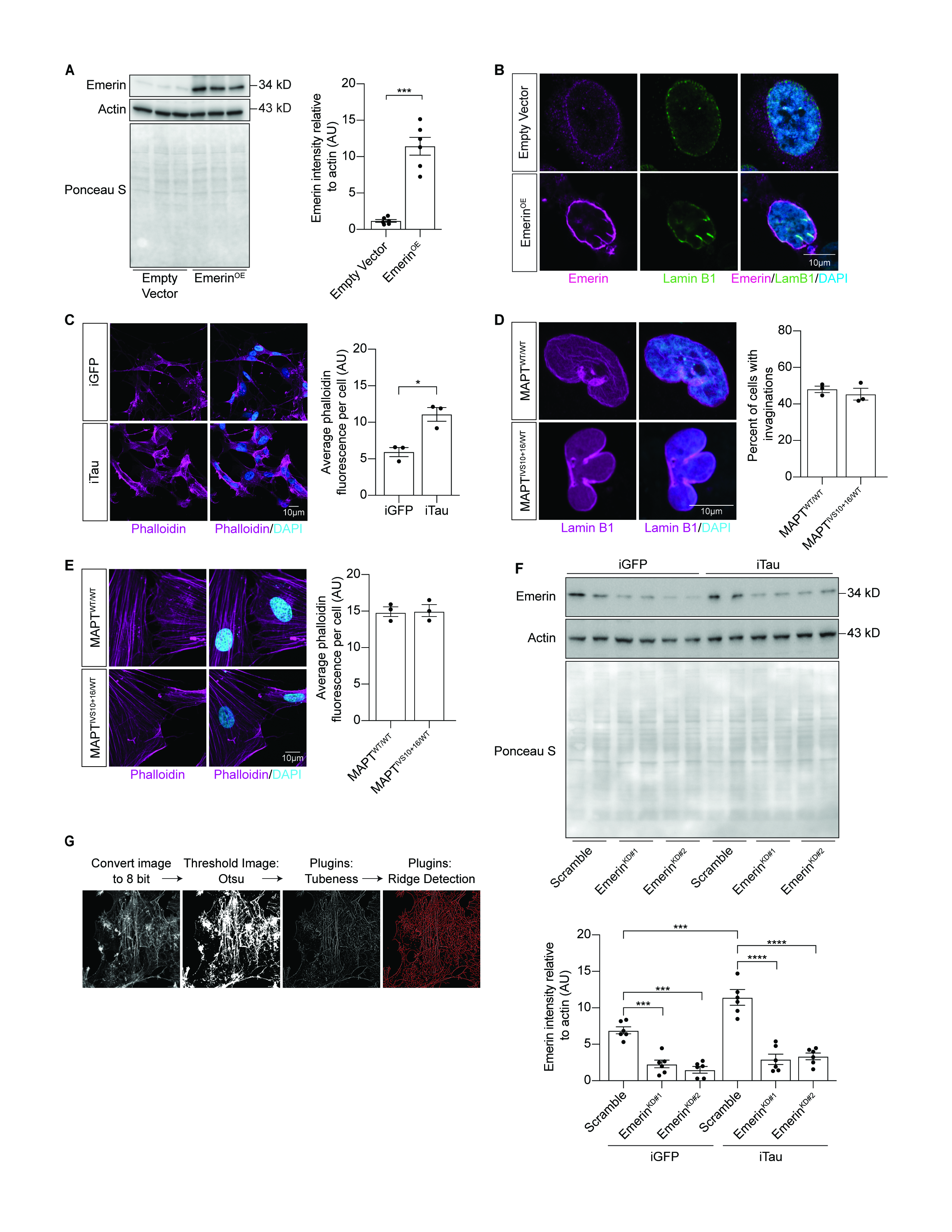

Supplement: Sohn Supp Fig 2.tif [file KNCL_A_2697135_SM0832.tif]

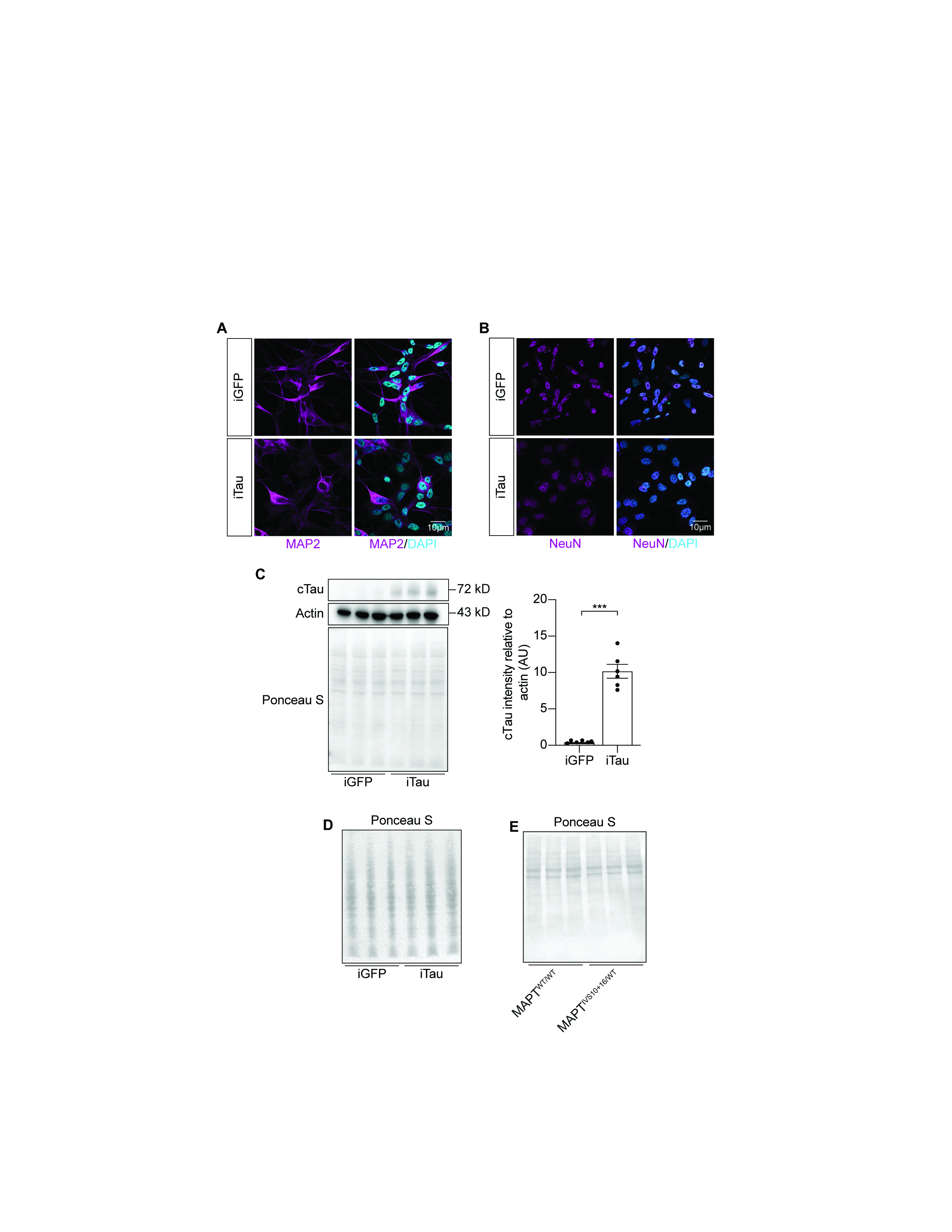

Supplement: Sohn Supp Fig 1.tif [file KNCL_A_2697135_SM0830.tif]
