# Supplementary material for: Elevation of the mechanically-sensitive e protein emerin links nuclear mechanotransduction to tau-induced cytoskeletal remodeling in neurons
Source: Nucleus. 2026 Jul 7;17(1):2697135. doi: 10.1080/19491034.2026.2697135 (PMC13349007; doi:10.1080/19491034.2026.2697135)
Supplement: 2026 Sohn Nucleus Supplemental Information.docx [file KNCL_A_2697135_SM0829.docx]

**SUPPLEMENTAL FIGURES**

**
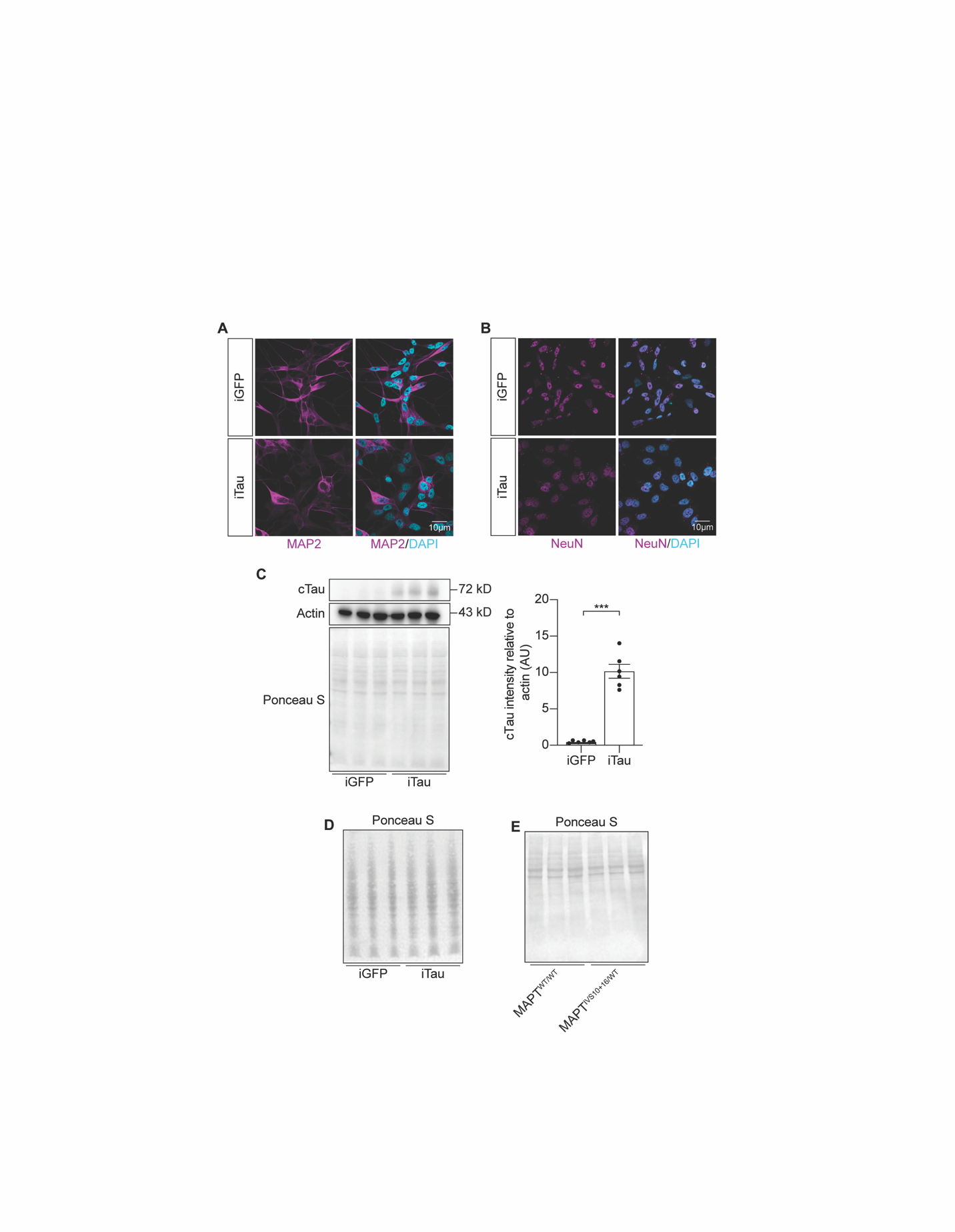
**

**Supplemental Figure 1**: **Neuronal identity analyses and tau induction in iGFP and iTau neurons.** One week of retinoic acid-mediated neuronal differentiation is sufficient for (**A**) MAP2 and (**B**) NeuN positivity in iGFP and iTau cells. (**C**) Western blot analysis of total tau protein levels after one week of retinoic acid-mediated neuronal differentiation and 24-hours of doxycycline treatment in iGFP and iTau neurons. Total protein levels as quantified by Ponceau S staining of membranes from Western blots presented in Fig. 1B (**D**) and Fig. 1E (**E**).


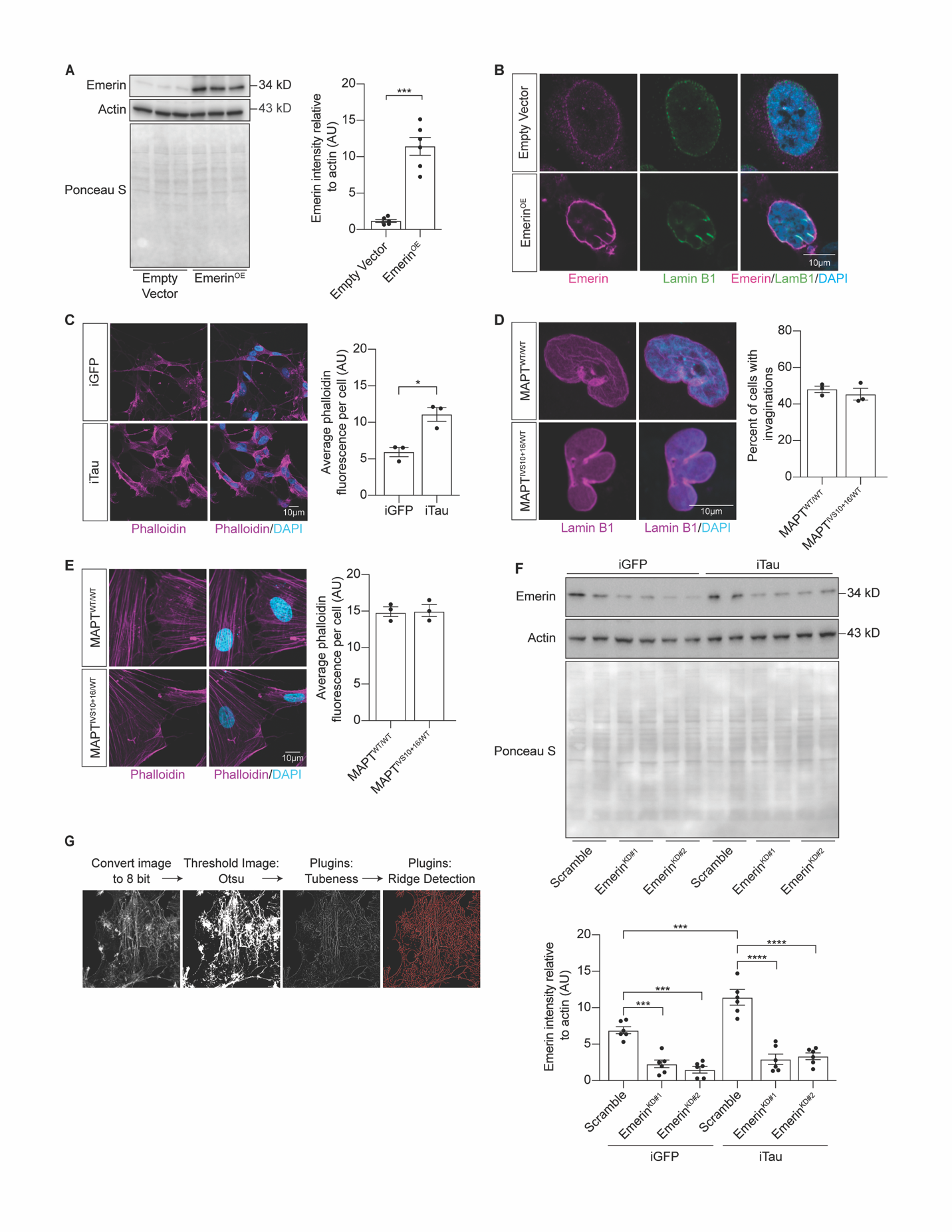


**Supplemental Figure 2**: **Consequences of genetic emerin manipulation in BE(2)-C-derived neurons and cellular phenotypes induced by pathogenic tau.** (**A**) BE(2)-C-derived neurons effectively overexpress emerin protein after transfection with an emerin overexpression plasmid based on Western blot. (**B**) Emerin localization in BE(2)-C neurons transfected with an emerin overexpression vector versus an empty expression vector. Visualization of nuclear envelope invaginations (**D**) and F-actin (**E**) in *MAPT^IVS10+16^* iPSC-derived neurons and isogenic control at eight weeks of neuronal differentiation. (**F**) Validation of emerin^KD^ based on Western blotting, with quantification. (**G**) Workflow for actin tract image analysis. n=3 biological replicates per group. t-test, *p≤0.05, ***p≤0.001, ****p≤0.0001. Error bars indicate SEM.


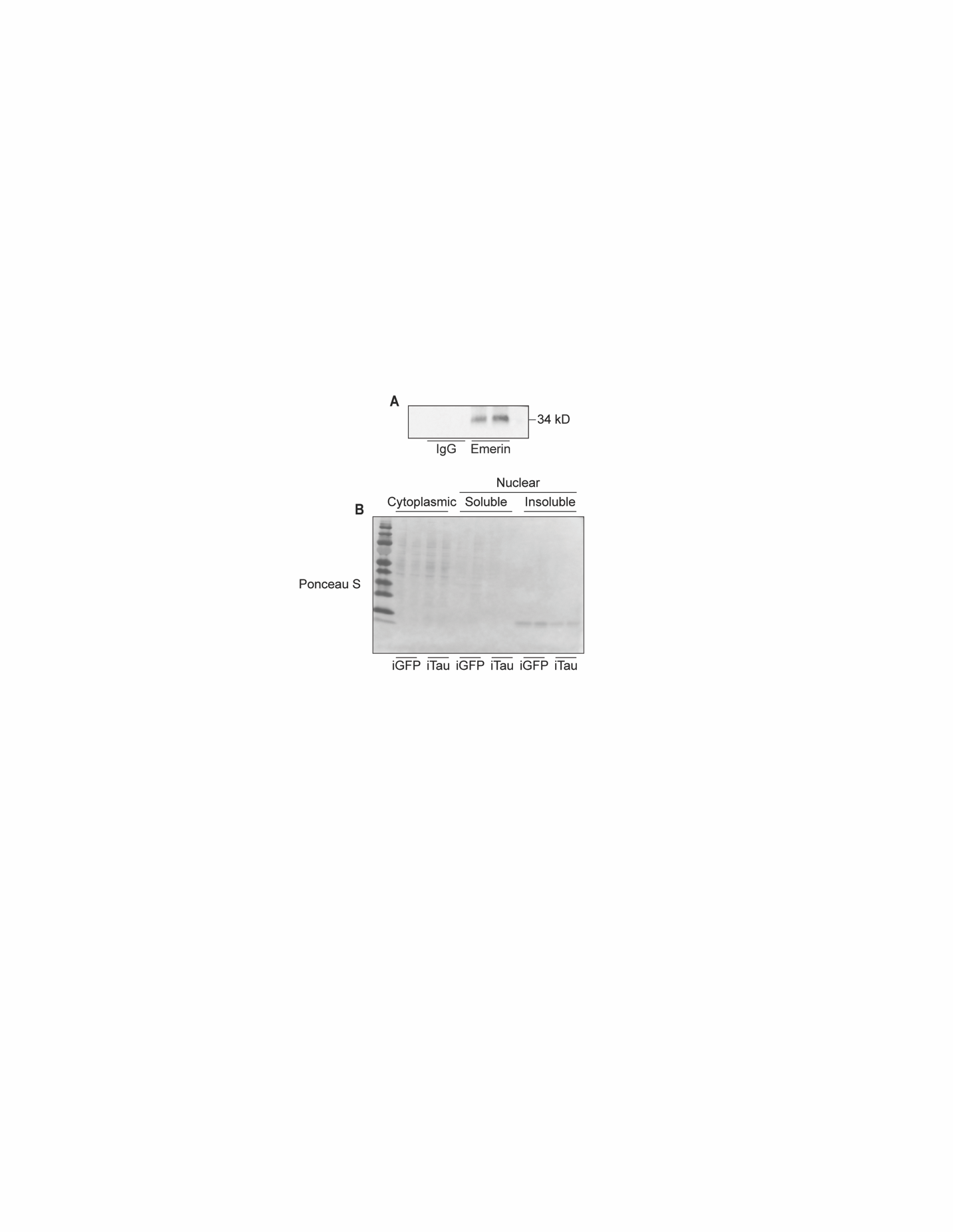


**Supplemental Figure 3**: **Immunoprecipitation of emerin and quantification of protein levels in cell fractionation analyses.** (**A**) Coomassie stain reveals effective immunoprecipitation of emerin using emerin antibody-loaded Dynabeads™ Protein-G; IgG-loaded Dynabeads serve as a negative control. (**B**) Ponceau S staining of Western blotting membrane from Fig. 4E.

**SUPPLEMENTAL TABLES**

| **Antibody** | **Catalog Number** | **Dilution** |
| --- | --- | --- |
| Anti-Emerin (rabbit polyclonal) | Abcam ab153718 | IHC: 1:500  WB: 1:5,000 |
| Anti-Emerin (rabbit polyclonal) | Abcam ab40688 | IP: 1 μg/μL |
| Anti-MAP2 (mouse monoclonal) | Invitrogen 13-1500 | IHC: 1:200 |
| Anti-Cleaved Caspase-3 (rabbit polyclonal) | Cell Signaling Technology 9661 | IHC: 1:400 |
| Anti-lamin B1 (mouse monoclonal) | Abcam ab8982 | IHC: 1:100 |
| Anti-lamin B1 (rabbit polyclonal) | Abcam ab16048 | WB: 1:500 |
| Anti-HSP90 (rabbit polyclonal) | Cell Signaling Technology 4874 | WB: 1:1000 |
| Anti-Actin (mouse monoclonal) | Developmental Studies Hybridoma Bank (DSHB) JLA20 | WB: 1:1000 |
| IgG Recombinant Rabbit Monoclonal Antibody | Invitrogen PSH04-42 | IP: 1 μg/μL |
| Anti-Histone3 H3 Rabbit Polyclonal Antibody | Abcam ab1791 | WB: 1:2000 |
| Goat Anti-Mouse IgG (H+L)-HRP | SouthernBiotech 1036-05 | WB: 1:20,000 |
| Goat Anti-Rabbit IgG (H+L)-HRP | SouthernBiotech 4030-05 | WB: 1:20,000 |
| Goat Anti-Rabbit IgG (H+L) 488 | ThermoFisher Scientific A-11008 | IHC: 1:200 |
| Goat Anti-Rabbit IgG (H+L) 555 | ThermoFisher Scientific A-21428 | IHC: 1:200 |
| Goat Anti-Mouse IgG (H+L) 488 | ThermoFisher Scientific A-11001 | IHC: 1:200 |
| Goat Anti-Mouse IgG (H+L) 555 | ThermoFisher Scientific A-21422 | IHC: 1:200 |

**Supplemental Table 1**: **Antibody sources and concentrations**

| **Dye** | **Catalog Number** | **Concentration** |
| --- | --- | --- |
| Acti-Stain 555 | Cytoskeleton Inc. PHDH1-A | 100 nM/coverslip |
| DAPI | ThermoFisher Scientific 62248 | 300 nM/coverslip |

**Supplemental Table 2**: **Cellular dyes, sources and concentrations**

**SUPPLEMENTAL DATA**

**Supplemental Data 1: DIA-MS analysis of iGFP and iTau cells and Metascape-based gene enrichment analysis.** n=3 biological replicates per condition.

**Supplemental Data 2: DIA-MS and Metascape-based gene enrichment analysis of emerin-interacting proteins in iGFP and iTau neurons.** n=3 biological replicates per condition.
